# Oligomerized, filamentous surface presentation of RANTES/CCL5 on vascular endothelial cells

**DOI:** 10.1038/srep09261

**Published:** 2015-03-20

**Authors:** Inger Øynebråten, Nicolas Barois, Trygve Bergeland, Axel M. Küchler, Oddmund Bakke, Guttorm Haraldsen

**Affiliations:** 1 Department of Pathology, Oslo University Hospital and University of Oslo, PO Box 4950 Nydalen, N-0424 Oslo, Norway; 2 Centre for Immune Regulation, University of Oslo, RikshospitaletPO Box 4950 Nydalen, N-0424 Oslo, Norway; 3 The Department of Biosciences, University of Oslo, PO Box 1041 Blindern, 0316 N-Oslo, Norway; 4 K. G. Jebsen Inflammation Research Centre, University of Oslo, RikshospitaletPO Box 4950 Nydalen, N-0424 Oslo, Norway

## Abstract

Vascular endothelial cells present luminal chemokines that arrest rolling leukocytes by activating integrins. It appears that several chemokines must form higher-order oligomers to elicit proper *in vivo* effects, as mutants restricted to forming dimers have lost the ability to recruit leukocytes to sites of inflammation. Here, we show for the first time that the chemokine RANTES/CCL5 binds to the surface of human endothelial cells in a regular filamentous pattern. Furthermore, the filaments bound to the surface in a heparan sulfate-dependent manner. By electron microscopy we observed labeling for RANTES on membrane projections as well as on the remaining plasma membrane. Mutant constructs of RANTES restricted either in binding to heparin, or in forming dimers or tetramers, appeared either in a granular, non-filamentous pattern or were not detectable on the cell surface. The RANTES filaments were also present after exposure to flow, suggesting that they can be present *in vivo.* Taken together with the lacking *in vivo* or *in vitro* effects of RANTES mutants, we suggest that the filamentous structures of RANTES may be of physiological importance in leukocyte recruitment.

At sites of inflammation, activated endothelial cells present luminal adhesion molecules and chemokines to recruit circulating leukocytes. A crucial step in this process is the arrest of rolling leukocytes that is triggered by chemokines and mediated by integrin activation^1^. Chemokines are a family of about 50 mainly secreted proteins which direct cellular migration through interaction with members of the seven transmembrane G protein coupled receptor family^2^,^3^,^4^. RANTES (regulated on activation, normal T cell expressed and secreted)/CCL5 is a highly basic, 68 amino acid, inflammatory chemokine that recruits a wide variety of leukocytes, including monocytes, granulocytes, T cells as well as mast cells and dendritic cells through interactions with the chemokine receptors CCR1, CCR3, and CCR5^4^.

Given that soluble chemokines would be rapidly washed away by the blood flow, chemokines are thought to be immobilized at the luminal surface of endothelial cells through low affinity interactions with sulfated glycosaminoglycan chains (GAGs) of proteoglycans^5^,^6^,^7^. Support for this hypothesis comes from the inhibited binding of chemokines to venules pretreated with heparinase^8^ as well as the reduced binding after targeted deletion of N-acetyl glucosamine N-deacetylase–N-sulfotransferase-1 required for the addition of sulfate to the heparan sulfate chains^9^. *In vitro*, several chemokines bind to GAGs such as heparin, heparan sulfate, chondroitin sulfate, and dermatan sulfate^5^,^10^. To this end, mutagenesis studies on RANTES have shown that the interaction of RANTES and heparin is mainly mediated through the highly basic BBXB motif ^44^RKNR^47^, located on the surface exposed 40s loop of the protein^11^,^12^. Replacement of these three basic residues with alanines (^44^AANA^47^) results in a variant with a substantially reduced heparin affinity^11^,^12^ and a complete inability to recruit inflammatory cells to the peritoneal cavity of mice^13^,^14^.

Another particular feature of RANTES is its propensity to oligomerize into higher-order complexes at high concentrations, high pH or in the presence of GAG^15^,^16^. For quite sometime, it was unclear if such oligomerization had any functional role or was merely an artifact of the high concentrations of protein used for structural studies. However, when mutants designed to remain monomeric or dimeric were injected in the peritoneal cavity, they failed to induce cellular recruitment, suggesting that in addition to GAG binding, oligomerization is required^13^,^17^. Several experiments indicate that GAG binding and oligomerization of chemokines are also functionally coupled^7^,^18^. For example, RANTES and several other chemokines may oligomerize on heparin beads. Dimeric forms of many chemokines have also been reported to have higher affinity for GAGs than their monomeric variants and GAG binding may involve, or induce, oligomeric structures larger than dimers. For example, although MCP-1/CCL2 forms a dimer in solution, a heparin octasaccharide shifts the equilibrium toward MCP-1 tetramers^19^.

In the present study, we have analysed the distribution of endogenous and recombinant RANTES on vascular endothelial cells. We show that secreted RANTES bound at the cell surface, not in a diffuse or aggregated pattern, but surprisingly as specific, well ordered filaments that elongated over time. The filamentous expression of RANTES was not found when expressing mutants that are restricted to form dimers or tetramers, or when expressing a mutant with reduced GAG affinity. These data add further support to the merging view that formation of higher order oligomers of RANTES is crucial to elicit its fully biological function in activation of leukocytes.

## Results

### RANTES is organized in filament-like structures on the endothelial cell surface

**Figure 1:**
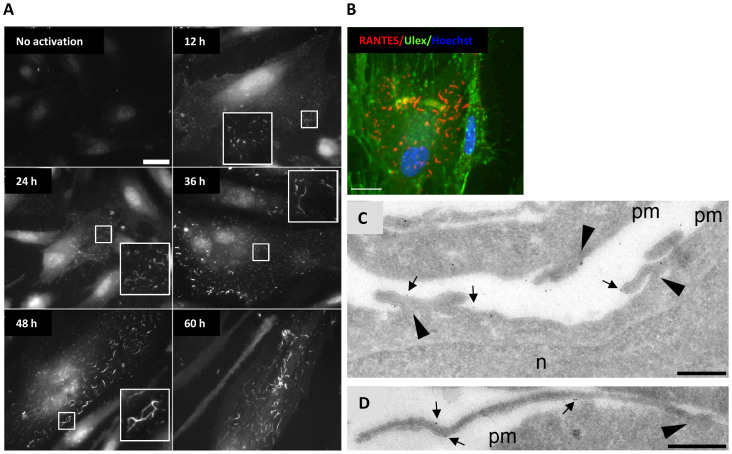
RANTES organizes in filaments on the cell surface and the filament length increases with incubation time in the presence of TNFα and IFNγ. (A) HUVECs were cultured in growth medium with 10 ng/ml TNFα and 1 ng/ml IFNγ for different time points (indicated in each image) before fixation and immunostaining with clone ID2/A12. Inserts show structure details at 3 × magnification. Scale bar, 10 μm. The images were acquired by widefield microscopy. (B) HUVECs were stimulated with 10 ng/ml TNFα and 1 ng/ml IFNγ for 30 h and then immunostained with a rabbit anti-RANTES antibody on ice to label only extracellular, surface associated RANTES. Ulex, a lectin, was utilized to label the surface of all HUVECs. Scale bar, 10 μm. Images were acquired by sequential scanning confocal microscopy. (C, D) HUVECs were stimulated with 10 ng/ml TNFα and 1 ng/ml IFNγ for 36 h and frozen for cryosectioning before immunogold detection of RANTES with a goat anti-RANTES antibody. The images show sections of the outer part of HUVECs, with membrane projections originating from the cell surface (arrowheads). Arrows indicate immunogold labelled RANTES. pm, plasma membrane; n, nucleus. Scale bars, 500 nm.

In a screen for chemokines that sort to the regulated secretory pathway in endothelial cells, we observed by means of immunofluorescent staining and confocal microscopy that RANTES was expressed in patterns distinct from those of other chemokines^20^,^21^ (Øynebråten *et al*., unpublished data). When HUVECs were cultured *in vitro* and stimulated with TNFα in combination with IFNγ before fixation and immunostaining, RANTES mainly localized in elongated, filamentous structures (Fig. 1A) and^20^. Five different antibodies towards RANTES were tested, and they all labeled elongated structures of RANTES. Analysis at different time points after exposing HUVECs to TNFα and IFNγ revealed that RANTES was distributed in puncta and short elongated structures after 12 h. In the course of analysis these structures elongated from an average length of 2 μm at 24 h to 15 μm after 60 h of stimulation (Fig. 1A). Based on these observations, we suggest that short structures of RANTES can develop into long filaments in cultures of endothelial cells activated by pro-inflammatory stimuli. To elucidate whether the filaments were present on the cell surface, we stained live HUVECs kept on ice, observing that RANTES filaments are subject to surface presentation on endothelial cells (Fig. 1B).

Several types of membrane projections have been described for endothelial cells^8^,^22^,^23^,^24^, and indeed, RANTES as well as IL-8/CXCL8 have been detected on microvillous-like extensions on the luminal endothelial cell surface^8^. We therefore asked whether RANTES filaments are associated with membrane projections in HUVECs. To this end, RANTES in cytokine-activated HUVECs was visualized by anti-RANTES antibody, gold-labeling, and electron microscopy. In these experiments, RANTES was observed both on HUVEC membrane projections and the remaining plasma membrane (Fig. 1C, 1D). Although there was a tendency of more labeling on the membrane projections, there was no significant difference in signal density between the two sites (Fig. S1).

### Filament formation does not depend on TNFα + IFNγ-stimulation

**Figure 2:**
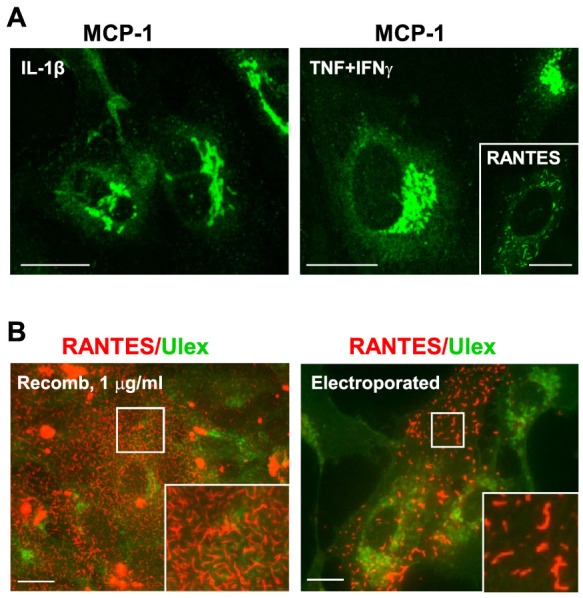
Filaments of RANTES form independently of TNFα and IFNγ-stimulation. (A) HUVECs were stimulated with 1 ng/ml IL-1β, or 10 ng/ml TNFα in combination with 1 ng/ml IFNγ for 24 h and immunostained with an antibody towards MCP-1. The insert shows immunostaining of RANTES from the same experiment, in HUVECs stimulated with 10 ng/ml TNFα + 1 ng/ml IFNγ. (B) Left image; HUVECs were incubated with 1 μg/ml recombinant RANTES before fixation and immunostaining. Right image; HUVECs were not stimulated but electroporated with a DNA plasmid encoding RANTES before fixation and immunostaining of RANTES with a rabbit anti-RANTES antibody. Labelling with ulex (green) was included to visualize individual cells. Scale bars, 10 μm. All images were acquired by confocal microscopy.

In agreement with a previous study^25^ we observed that RANTES was most strongly induced in HUVECs by simultaneous stimulation with TNFα and IFNγ^20^. Because we did not observe filamentous organization of chemokines in resting or IL-1β stimulated-HUVECs^20^,^21^ we asked if the particular expression pattern of RANTES might be associated with the activation program induced by TNFα + IFNγ-stimulation. However, MCP-1/CCL2 showed a non-filamentous distribution/pattern regardless of being labeled in HUVECs stimulated with IL-1β or with TNFα + IFNγ (Fig. 2A), indicating that TNFα + IFNγ-stimulation is generally not sufficient to induce a filamentous organization of chemokines. On the other hand, when recombinant RANTES was added to cultures of unstimulated HUVECs, RANTES organized in filaments. The filaments were numerous following incubation with 1 μg/ml of RANTES (Fig. 2B, left panel). In addition, some patches of RANTES were observed, probably formed because of the high RANTES concentration (Fig. 2B, left panel). Recombinant RANTES in concentrations 1 and 10 ng/ml failed to induce filaments (data not shown), suggesting that concentrations above 10 ng/ml are necessary for filament formation when RANTES derive from an extracellular source. Electroporation with a DNA plasmid encoding RANTES, also resulted in filamentous organization of the protein (Fig. 2B, right panel). Thus, filamentous organization is a property of RANTES which does not depend on TNFα + IFNγ-stimulation.

### Extracellular molecules can polymerize RANTES

**Figure 3:**
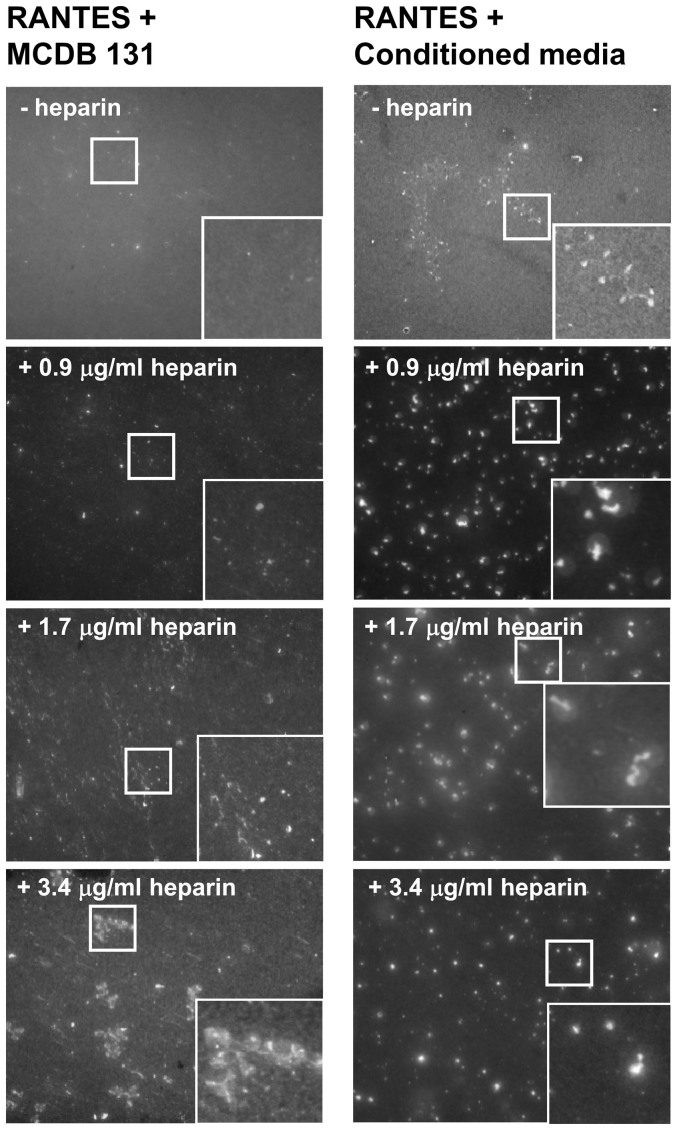
RANTES is dependent on other molecules to form organized structures. RANTES (1 μg/ml) was incubated either in cell growth medium without serum or in conditioned cell growth medium containing serum. The conditioned medium was harvested from unstimulated cultures of HUVECs. Heparin was added and its final concentration is indicated in each image. After 35 h, the samples were fixed and immunostained with a rabbit antibody toward RANTES. The images were acquired by widefield microscopy. Inserts show high magnification of squared areas. Scale bars, 10 μm.

We next examined whether RANTES alone can polymerize and form filaments or whether other molecules are necessary (Fig. 3). First, we incubated RANTES (1 μg/ml) with cell growth medium (MCDB131) without serum, or with conditioned media harvested from unstimulated HUVECs. Based on the experiments shown in Fig. 1A where we observed that the filaments grew in size with time, we chose to incubate all samples for 35 h. Chemokines typically bind to GAGs via positively charged amino acids, therefore, we added the GAG heparin in different concentrations to some of the wells. In the first series, we added increasing concentrations of heparin to cell growth medium without serum, observing at low and medium concentrations of heparin (0.9 and 1.7 μg/ml, respectively) no evidence of filament formation of RANTES (Fig. 3, left panel). However, following incubation with the highest concentration of heparin (3.4 μg/ml), RANTES organized in a structured pattern that nevertheless differed from that observed for RANTES in HUVEC cultures (Fig. 3, left panel, lower image). In the next series we added increasing concentrations of heparin to conditioned media, observing even in the absence of heparin that RANTES was distributed in an organized and distinct pattern (Fig. 3, right panel, upper image). When increasing the concentration of heparin (1.7 μg/ml) we observed that RANTES was organized in a pattern reminiscent of the RANTES filaments in HUVECs, and when reaching the highest level (3.4 μg/ml), RANTES was found in puncta (Fig. 3, right panel, lower image). Taken together, these experiments suggest that RANTES cannot alone organize into filaments and instead depends on helper or scaffold molecules. In addition, RANTES can organize into different patterns, likely depending on the type of molecules that are present.

### Heparan sulfate is involved in cell surface immobilization of RANTES

**Figure 4:**
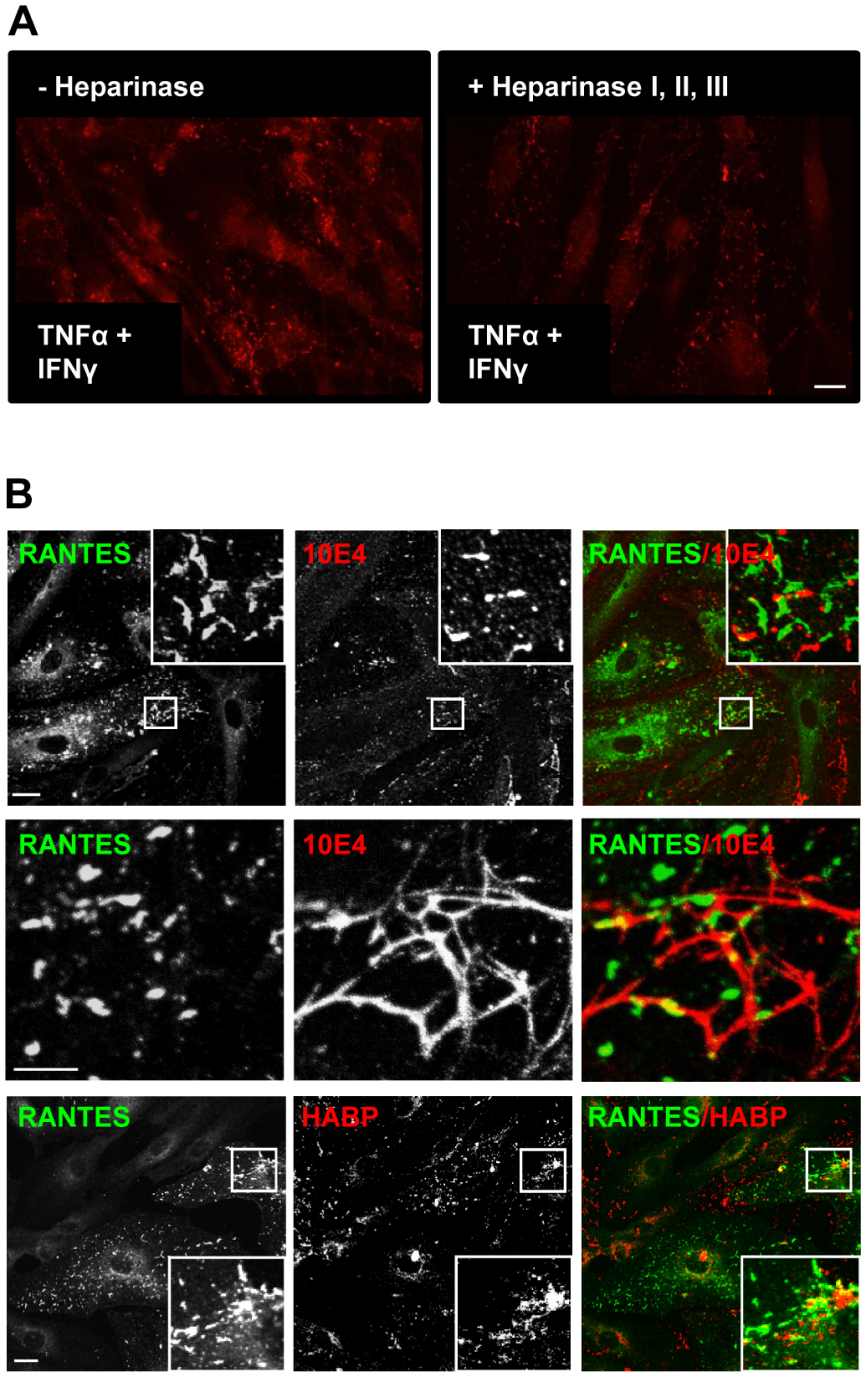
RANTES is immobilized to the cell surface via heparan sulfate. (A) HUVECs were stimulated for 48 h with 10 ng/ml TNFα + 1 ng/ml IFNγ before half of the samples were incubated with a mixture of heparinase I, II, and III (0.5 U/ml) for 2 h. Next, the cells were fixed and immunolabelled with clone ID2/A12 and analyzed by widefield microscopy. Scale bars, 50 μm. (B) HUVECs stimulated with 10 ng/ml TNFα and 1 ng/ml IFNγ were immunostained with antibodies towards RANTES (rabbit anti-RANTES antibody) and the heparan sulfate epitope 10E4. Alternatively, biotinylated hyaluronan binding protein (HABP) was used to label hyaluronan. The antibody towards 10E4 labelled elongated structures in HUVECs (upper panel) and long structures at cell borders or between cells (middle panel). Middle panel is a high magnification from an original 100 × picture. Biotinylated HABP labelled irregular clusters in HUVECs, and large, round structures between cells (lower panel). The samples were analyzed following sequential scanning confocal microscopy. Corner insets show high magnification of framed areas. Scale bars, 10 μm.

It is well established that GAGs can immobilize chemokines on cell surfaces^5^,^9^,^26^,^27^. Because of this knowledge, and the finding that incubation of RANTES with heparin leads to formation of organized structures of RANTES (Fig. 3, left panel), we wanted to examine whether GAGs in HUVEC cultures affect the generation of filaments. Monolayers of HUVECs were stimulated with TNFα + IFNγ before treatment with a mixture of heparinase I, II, and III, and immunostaining for RANTES. Compared to the control sample (Fig. 4A, left image), heparinase treatment reduced the intensity of the RANTES signal by 55% (Fig. 4A, right image), suggesting that heparan sulfate is involved in surface binding. Aiming to verify this finding by use of another approach, we stained TNFα + IFNγ-stimulated HUVECs with an antibody recognizing the epitope 10E4, a common epitope in heparan sulfate^28^,^29^,^30^. Immunostaining revealed different patterns of heparan sulfate distribution (Fig. 4B). One pattern was cell-associated, showing elongated structures similar to and overlapping with those of RANTES (Fig. 4B, upper panel), but overt co-localization was not observed. Another pattern appeared to be present at the cell border or between cells. At these sites, the antibody labeled long thread-like structures, and filaments of RANTES was observed along these structures (Fig. 4B, middle panel, and Fig. S2). Finally, because incubation of RANTES in conditioned media indicated that large scaffold molecules could organize RANTES (Fig. 3, right panel; upper image), we also labeled HUVEC cultures with biotinylated hyaluronan binding protein (bio-HABP). HAPB shows high affinity for a decasaccharide unit of hyaluronan, which can form large polymers up to 20,000 kDa that organize into a wide variety of molecular architectures including fibrils and cable-like structures^31^. Except from some regions with large clusters of RANTES and HABP associated with HUVECs, we observed no co-localization between RANTES and HABP (Fig. 4B, lower panel). Between cells, HABP was found in large, round structures that were somewhat irregular and negative for RANTES (data not shown). Based on these experiments, we concluded that heparan sulfate molecules bind RANTES in HUVEC cultures, but the importance of other molecules for immobilization of RANTES cannot be excluded.

### Filamentous distribution of RANTES depends on the ability to form higher-order oligomers

RANTES can self-associate and form higher-order oligomers in a concentration-dependent manner^5^,^32^. In contrast, two mutants of RANTES, E26A and E66A/E66S, show strongly reduced ability of such oligomer formation, and are instead restricted to form tetramers (E26A) and dimers (E66A/E66S)^16^,^32^,^33^. Furthermore, the 40s loop (^44^RKNR^47^) is suggested to be important for oligomerization as R44 may exert stabilization forces to the dimer interface, and R47 is shown to interact with the neighbouring molecule in RANTES structures^16^,^34^.

**Figure 5:**
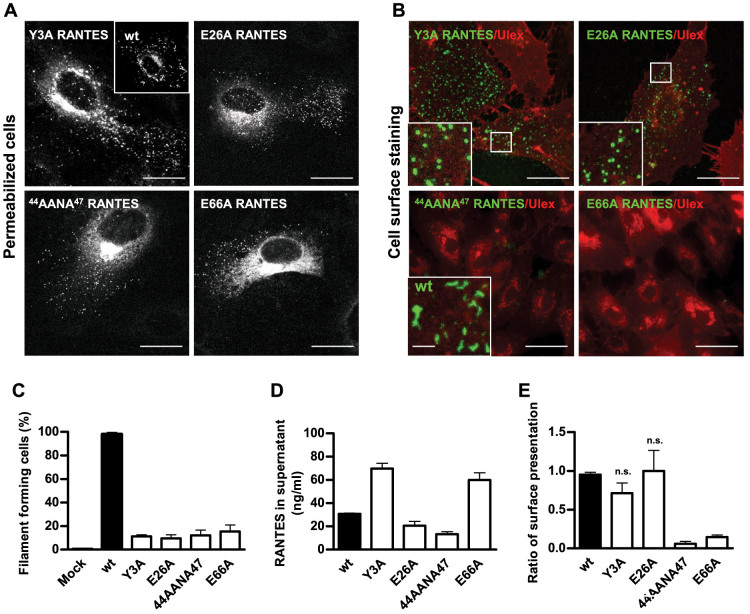
Oligomerization-deficient mutants of RANTES show distinct morphology and localization compared to the wild type (wt). (A) HUVECs were electroporated with DNA plasmids encoding wtRANTES or oligomerization-deficient mutants and cultivated for 24 h before fixation. The cells were permeabilized to label RANTES present intracellularly and on the surface with rabbit anti-RANTES antibody. Images were acquired by confocal microscopy. Corner inset shows a cell electroporated with wtRANTES-encoding plasmid. Scale bars, 10 μm. (B) The experiment was performed as indicated in A, but labelling was performed on live HUVECs kept on ice to detect cell surface-associated RANTES with rabbit anti-RANTES antibody. Labelling with the lectin ulex was used to visualize individual cells. Images were acquired by sequential scanning confocal microscopy. Corner insets show high magnification of framed areas or high magnification of a cell electroporated with DNA encoding wtRANTES. Scale bars, 10 μm (wt, scale bar = 5 μm). (C) HUVECs were treated as described in A, and the number of RANTES-positive and filament-forming cells were counted. The graph presents mean values of percentage of filament-forming cells related to the total number of RANTES-positive cells. 55–80 cells were evaluated for each construct in one experiment (n = 3 experiments). Error bars indicate SEM. The mutants generated a significantly lower percentage of filament-forming cells than the wt, p < 0.0001. (D) HUVECs electroporated with DNA encoding the indicated constructs were incubated for 30 h before supernatants were harvested. The amount of RANTES in supernatants was quantified by ELISA utilizing recombinant RANTES as standard. The mutants were present in amounts that differed significantly from the wt, p < 0.0001. Error bars indicate SEM, n = 3–6 experiments. (E) HUVECs were treated as indicated in A, but half of the samples were kept live on ice during labelling to indicate cell surface-associated RANTES. Labelling with ulex was used to visualize individual cells. The graph shows mean values of the percentage of filament forming-cells related to the total number of RANTES-positive cells. 55–80 cells were evaluated for each construct in one experiment (n = 3 experiments). Error bars indicate SEM. Surface presentation of ^44^AANA^47^ and E66A differed significantly from that of the wt, p < 0.0001. n.s., not significant.

To elucidate whether the filamentous pattern of RANTES might be related to the properties of oligomer formation we electroporated HUVECs with DNA plasmids encoding E26A, ^44^AANA^47^, or E66A. We also examined the mutant Y3A which shows *in vivo* properties similar to ^44^AANA^47^ but has unknown oligomerization status^34^. Microscopy after electroporation and immunostaining of the mutants E26A, ^44^AANA^47^, and E66A revealed a pattern substantially different from that of wild type (wt) RANTES (Fig. 5A). The mutants mainly appeared in small granular structures as well as in the Golgi. ^44^AANA^47^ and E66A were also apparent throughout the cytoplasm, reminiscent of endoplasmic reticulum staining (Fig. 5A). Only 10–17% of the mutant-expressing HUVECs showed filamentous RANTES compared to 98% for the wt (Fig. 5C). The mutant Y3A distributed in a pattern similar to that of the other mutants suggesting that it had lost the ability to form higher-order oligomers (Fig. 5A).

To exclude that the dramatic reduction of filament formation was caused by low RANTES expression, we performed several control experiments. First, we compared the intensity of the signals for the immunolabeled mutants towards that of the wt by recording images at identical exposure times. By this comparison, we observed that the signal intensity of filament-forming wtRANTES varied and that it was present both at lower and higher levels than the signal of the mutants on a per cell basis. Even cells expressing wtRANTES at low levels (based on a very weak signal) showed a filamentous RANTES pattern (data not shown). Moreover, we electroporated HUVECs with low amounts of plasmid DNA encoding wtRANTES (4 μg compared to 20 μg in the standard protocol) which resulted in a dramatic reduction in protein expression and fewer positive cells. Despite the low level, wtRANTES organized in filaments (data not shown). Finally, we performed ELISA on supernatants harvested from HUVECs electroporated with plasmid DNA encoding the various constructs. Both Y3A and E66A were present in higher amounts than wt, whereas E26A and ^44^AANA^47^ were found in approximately two-thirds and half the amount, respectively, of that measured for wt (Fig. 5D). Altogether, these data confirmed that the different patterns were not a result of various expression levels per cell or by the culture. Therefore, our findings clearly suggest that organization into filaments depends on the ability to form higher-order oligomers.

Next, to examine whether the mutant constructs of RANTES were present on the endothelial cell surface or restricted to intracellular compartments, we immunolabelled live HUVECs kept on ice and compared the pattern towards that of fixed and permeabilized cells. As shown in Fig. 5B (upper panel), the mutants Y3A and E26A distributed in a granular pattern throughout the HUVEC surface similar to that observed in permeabilized cells, suggesting that their binding to the surface was not disrupted. We also calculated the ratio between the number of cells expressing the mutant on the surface and the number of cells expressing the mutant after permeabilization (Fig. 5E). By this analysis we found that the ability of surface expression was not significantly affected for E26A or Y3A in comparison to the wt. In contrast, we found dramatically reduced surface expression for ^44^AANA^47^ and E66A.

### RANTES does not co-localize with ICAM-1

**Figure 6:**
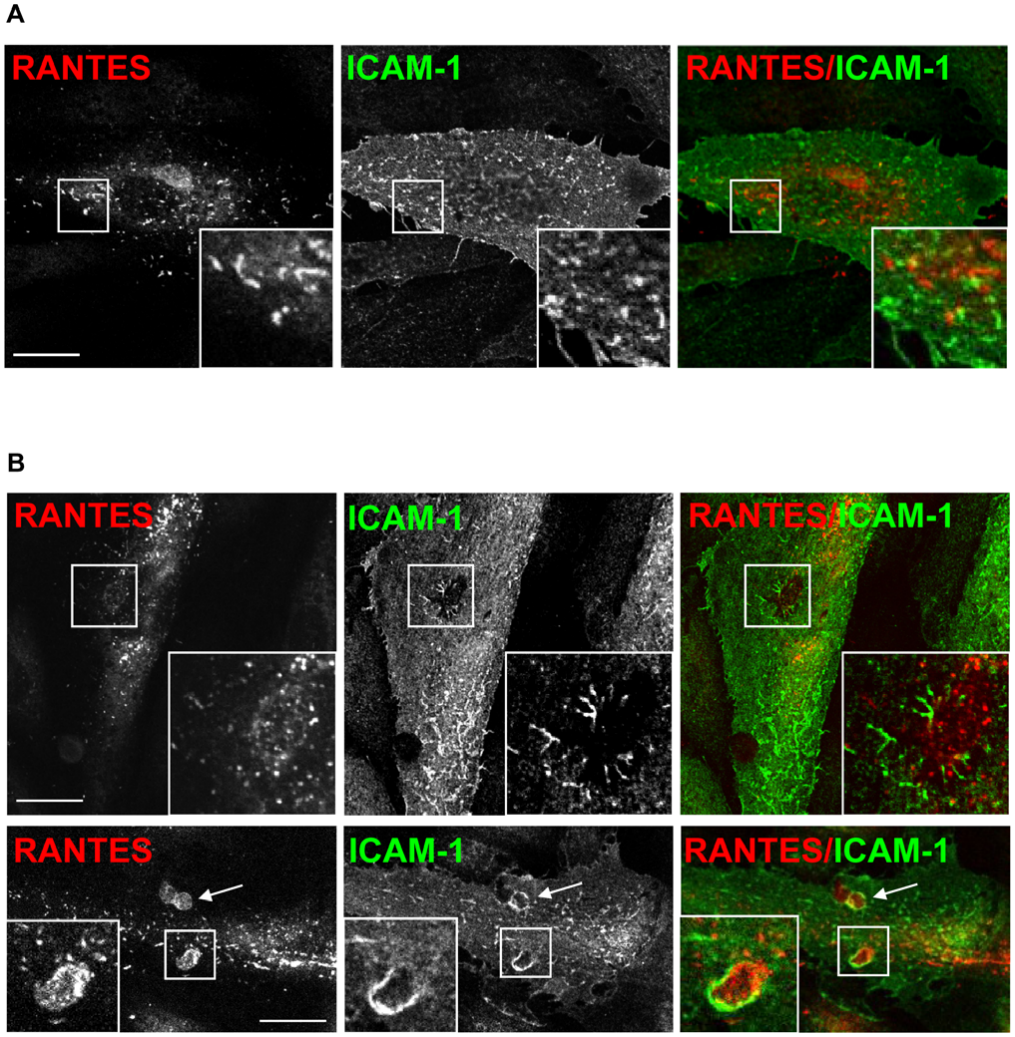
RANTES shows distinct localization from that of ICAM-1. (A) Localization of RANTES compared to that of ICAM-1 after electroporation of HUVECs with plasmid DNA encoding wtRANTES and stimulation with TNFα before fixation, permeabilization and immunolabelling. Corner insets show high magnification of framed areas. Scale bar, 10 μm. (B) HUVECs were treated as indicated in (A) before incubation with MCP-1, followed by addition of peripheral blood mononuclear cells for 20 min followed by fixation, permeabilization, and immunolabelling. Corner insets show high magnification of framed areas, which are areas where one leukocyte has transmigrated. Arrows in the lower panel indicate two leukocytes that may have transmigrated. Original magnification in all panels, × 100. Scale bars, 10 μm. A rabbit anti-RANTES antibody was utilized in immunolabelling of RANTES. All samples were analyzed by use of sequential scanning confocal microscopy.

Pre-existing tetraspanin-enriched microdomains (TEMs) containing adhesion receptors such as ICAM-1, VCAM-1, and CD44 are suggested to function as adhesive platforms on the endothelial cell surface^35^,^36^. We wanted to examine whether RANTES localized in such platforms, using ICAM-1 as a marker. Consistent with previous findings^37^, activation of HUVECs with TNFα or TNFα combined with IFNγ upregulated the ICAM-1 expression in all cells. Interestingly, the brightest cells had a speckled surface expression of ICAM-1 reminiscent of the pattern seen for RANTES. However, paired immunostaining for RANTES and ICAM-1 showed spots of overlapping signal but generally no co-localization (Fig. 6A). Previous studies have demonstrated an impressive redistribution of ICAM-1 during para- or transcellular leukocyte migration across the endothelium. ICAM-1 was enriched in vertical microvilli-like projections that embraced the leukocyte and drove redistribution of their integrins into linear tracks parallel to the direction of diapedesis^35^,^38^,^39^. To elucidate whether RANTES distributed together with ICAM-1 into such a docking structure or transmigratory cup upon leukocyte addition, we reproduced the experiments by transfecting HUVECs to express RANTES, exposing them to TNFα, and adding peripheral blood mononuclear cells to such monolayers 20 min before fixation. While ICAM-1 was indeed observed in projections surrounding the leukocyte, the same projections were negative for RANTES (Fig. 6B). However, RANTES could be observed close to ICAM-1. RANTES was also observed at sites of docking or transmigration, but the strongest signal was typically observed in areas which were weak or negative for ICAM-1 (Fig. 6B, lower panel). Taken together, these experiments suggest that RANTES is not present on ICAM-1 positive microvillous-like projections. In addition, because RANTES did not show a regular presence close to ICAM-1 on the surface, we suggest that RANTES is not a crucial molecule of the endothelial adhesive platform.

### The RANTES filaments prevail flow forces

**Figure 7:**
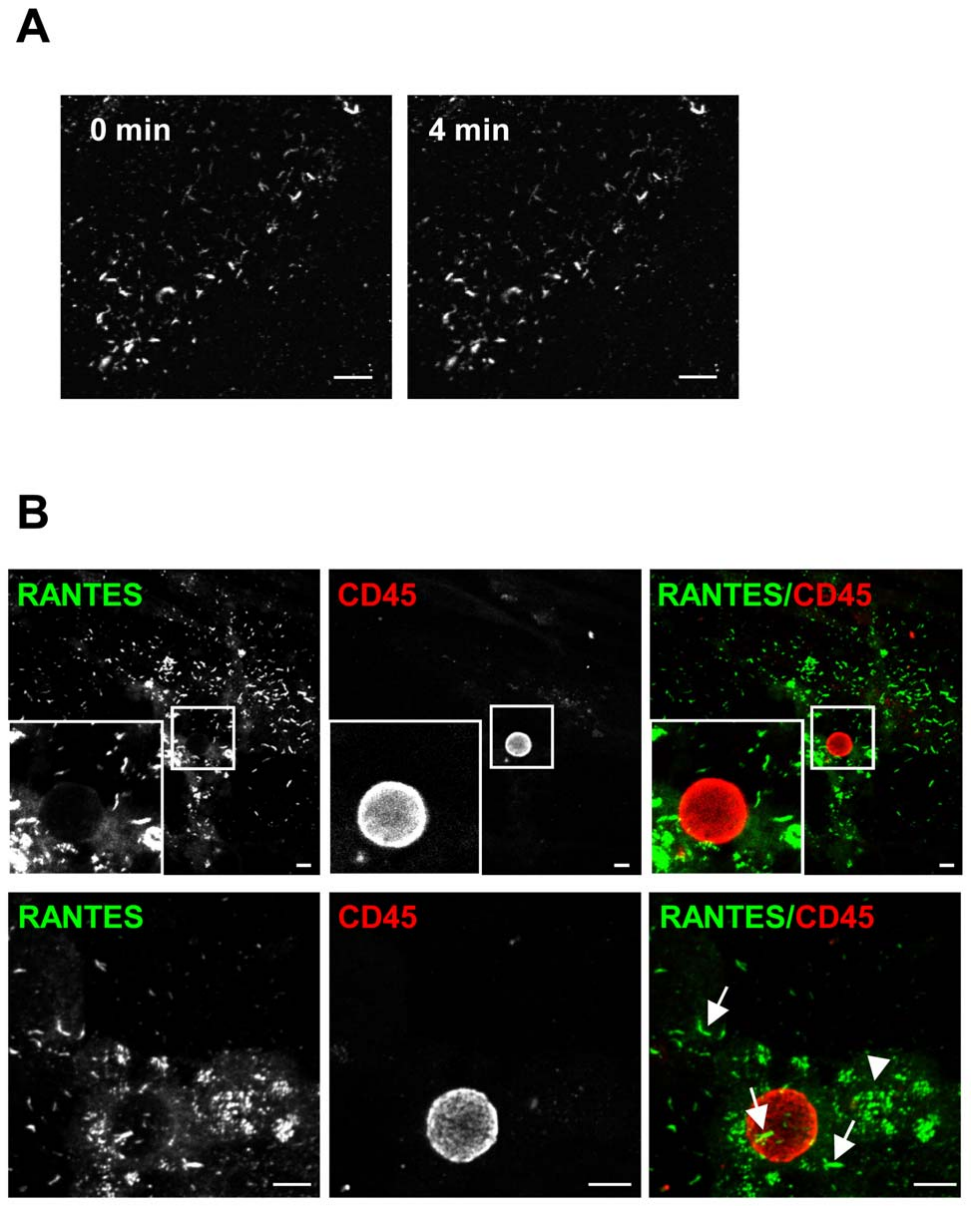
RANTES filaments are present after exposure to shear stress. (A) HUVECs were electroporated with DNA encoding RANTES, cultivated on cover slips before stimulation with TNFα + IFNγ for 30 h. RANTES was labeled with an anti-RANTES antibody (clone 21418), and the cover slips were mounted in a laminar flow chamber. The flow rate was adjusted to mimic vessel wall shear stress of 1 dyne/cm^2^. The images were acquired by confocal microscopy before (left image, 0 min) and after 4 min with exposure to flow forces (right image, 4 min). (B) HUVECs were treated as described in (A) except that they were not labeled with anti-RANTES antibody before exposure to flow. Human peripheral blood mononuclear cells were labeled with an anti-CD45 antibody, resuspended in medium and applied by a pump to the flow chamber (1 dyne/cm^2^). After 10 min, the cells were labeled with a rabbit anti-RANTES antibody. Images were acquired by sequential scanning confocal microscopy. Arrows and arrow heads indicate RANTES filaments and RANTES positive platelets, respectively. Original magnification in all panels, × 100. Scale bars, 10 μm.

Because of the blood flow, molecules on the endothelial cell surface or at sites of vascular injury may be exposed to shear stress. To examine whether RANTES filaments can persist or will be disrupted by shear forces, we electroporated HUVECs with DNA encoding RANTES, cultivated the cells on cover slips before exposing them to TNFα + IFNγ and mounting them in a laminar flow chamber. We chose to expose the HUVECs to flow mimicking shear stress of 1 dyne/cm^2^ as the force has been shown to support adhesion of both monocytes and lymphocytes^40^,^41^. Four minutes after exposure to flow, the RANTES filaments were still present, suggesting that they can exist *in vivo* (Fig. 7A). In other experiments, peripheral blood mononuclear cells visualized by anti-CD45 staining were added under flow conditions. Although RANTES filaments and RANTES-positive platelets could be observed in close proximity to adhering mononuclear leukocytes, we could not observe RANTES-positive structures resembling a transmigratory cup (Fig. 7B). Taken together, the RANTES filaments with or without exposure to flow forces appeared to be similar, suggesting that they can be present *in vivo*.

## Discussion

Several lines of evidence support a role for higher-order, oligomerized chemokines in leukocyte recruitment. First, wtRANTES but not disaggregated mutants, recruits leukocytes to the peritoneal cavity^13^,^14^. Second, disaggregated mutants of RANTES fail to support leukocyte arrest to cultured endothelial cells under flow conditions^42^, and some of these mutants are powerful anti-inflammatory agents^14^,^34^,^43^. Taken together, these findings point to a possible role for oligomerized RANTES at the endothelial cell surface. Here we show for the first time morphological evidence that RANTES indeed binds to the endothelial cell surface in a regular, filamentous pattern. This depends on the oligomerization state of RANTES as all disaggregated mutants (identical or complementary to those tested under flow or *in vivo)* failed to appear as filaments on cultured endothelial cells.

Our observation that the mutant ^44^AANA^47^ did not form filaments (likely because it failed to bind the endothelial cell surface) fits well with its rapid appearance in peripheral blood after intraperitoneal injection^14^, indicating that the mutant is not trapped in the tissue/extracellular matrix as efficiently as the wt and/or does not bind to the endothelial cell surface after abluminal to luminal transcytosis. This hypothesis would also be compatible with the independent observation that recombinant ^44^AANA^47^-RANTES added to cultures of human microvascular endothelial cells does not bind to the cell surface^42^. Taken together with the observation that the number of filaments was reduced after heparinase treatment, these data are consistent with the view that ^44^RKNR^47^ constitutes the principal GAG-binding site^44^, and that binding to GAG is crucial for immobilization to the endothelial cell surface^5^,^8^,^13^.

In our experiments, filament formation generated by the tetramer-restricted mutant E26A was reduced by more than 90% compared to the wt. Following injection into the mouse peritonitis model, E26A and wtRANTES appeared to be equally efficient in recruitment of cells, and it was concluded that the smallest leukocyte-recruiting form of RANTES is a tetramer^13^. This conclusion was based upon counts of the total number of cells in peritoneal lavage harvested 18 hours after injection of wt and E26A protein^13^. However, when considering different steps of the leukocyte extravasation cascade *in vitro* under flow conditions, the E26A mutant showed reduced potency compared to the wtRANTES. The mutation E26A reduced the number of monocytes that arrested to the endothelial monolayer, and the authors suggested that RANTES oligomers are required to bridge surface-bound RANTES and CCR1^42^. Thus, in addition to the inability to form filaments, the E26A mutant shows reduced potency compared to that of wtRANTES in “isolated” steps of the extravasation of leukocytes.

The mutant Y3A shares with the heparin-deficient mutant ^44^AANA^47^ the inability to recruit leukocytes in inflammation and the ability to inhibit cell recruitment induced by wtRANTES^34^. In contrast to the ^44^AANA^47^ variant, Y3A binds heparin. We found that Y3A, similar to E26A, appeared in a granular pattern on the endothelial cell surface, indicating that its ability to form oligomers is abolished. Finally, the E66A mutant which forms dimers, was detected intracellularly and was secreted in similar amounts to that of Y3A. Recombinant E66A was reported to bind to the surface of human microvascular endothelial cells^42^ whereas this was not the case in our study. Binding of RANTES molecules to heparan sulfate have been suggested to occur by positive cooperation, meaning that the binding affinity for the second RANTES molecule is higher than for the first one^45^. Given that the model of positive cooperation applies for binding of RANTES units larger than dimers, the affinity for E66A could be lower than for the other constructs and may explain its reduced surface presentation.

Interestingly, Wang *et al*. proposed a model for how RANTES organizes into oligomers based on detailed structural analyses^16^: The RANTES dimer is the building block, and long linear polymeric chains can form by contacts between residues of the second β-strand and residues at the C-terminal helix from one monomer of a dimer and similar residues in the neighbouring dimer. Consistent with the reduced ability of E26A and E66A to oligomerize and form filaments, both E26 and E66 appear to exert stabilization forces on the interaction between RANTES dimers. Wang *et al*. observed long oligomers at pH 7, and suggested that binding to sulfated GAGs would further promote length, as negatively charged GAG can neutralize electric repulsion forces between RANTES dimers^16^. In our cell cultures, presence of RANTES filaments was at least partially dependent on heparan sulfate as there were fewer filaments following heparinase treatment. A crucial role for GAGs in promoting filament length, was supported by the finding that in the absence of cells, heparin was needed for filaments to appear.

During the initial events of leukocyte extravasation, membrane projections would probably increase the accessibility of the endothelium towards that of the leukocyte. Luminal endothelial membrane projections have been given names such as microfolds, microvilli, filopodia, protrusions, and nanotubes^8^,^22^,^23^,^24^, and some of these are likely different structures with distinct functions. Similar to what was recently reported^24^, we observed long membrane projections lateral to the endothelial cell surface as well as shorter membrane projections. Interestingly, Whittall *et al*. found that leukocytes interacted with both the long and short projections^24^. By immunogold labeling of RANTES and electron microscopy, we observed a tendency of more labeling on the membrane projections than on the remaining plasma membrane. Unfortunately, the lower staining intensity obtained by immunogold labeling than by immunofluorescence, combined with the lost orientation of cells after scraping, did not allow us to draw firm conclusions on where the RANTES filaments localized at an ultrastructural level. Nevertheless, chemokines localized to membrane projections have been reported by others. For example, IL-8 immunoreactivity was detected at tenfold higher levels on vascular luminal projections than on the remaining plasma membrane^8^. RANTES was also detected on such membrane projections^8^.

Upon binding to leukocyte chemokine receptors, chemokines can trigger complex signaling transduction cascades leading to activation of integrins and, ultimately, to adhesion via binding to adhesion molecules such as ICAM-1^46^. As this occurs rapidly and integrin activation is a reversible process, co-localization of RANTES and ICAM-1 could increase the possibility for the activated leukocyte to interact with ICAM-1. However, we were unable to observe overt co-localization both during the process of peripheral blood mononuclear cell extravasation and in the absence of leukocytes. By scanning electron microscopy we observed membrane projections resembling microfolds or small villi on the HUVEC surface (Øynebråten *et al*., unpublished data). This pattern was reminiscent of the ICAM-1 distribution observed after immunofluorescence, and our immunofluorescent data are very similar to what has been reported by others with ICAM-1 being present on microvilli or microvilli-like projections^38^,^39^,^47^,^48^. Taken together, our data suggest that RANTES and ICAM-1 are present at different membrane sites.

We have stained for numerous chemokines after cytokine-stimulation or transfection of chemokine-encoding DNA into cultures of endothelial cells^20^,^21^ (Øynebråten *et al*., unpublished data). In our studies the filaments were unique to RANTES, and due to high positive charge, it is proposed that RANTES is hindered from forming the more common organization of chemokine high order oligomers, *i.e*. globular complexes^7^,^16^. However, it cannot be excluded that other chemokines than those we tested, for example MIP-1α/CCL3 and MIP-1β/CCL4, can form such elongated structures, although their charge and their binding to GAGs differ from that of RANTES^16^,^49^. RANTES organized in different structures depending on the conditions in the well (presence of cells, heparin, conditioned media). Therefore, our data suggest that RANTES binding molecules are crucial for the organization of RANTES and its presentation to chemokine receptors. Given that filaments are formed *in vivo*, our data imply that presence of filaments may vary between types of endothelial cells, the tissue site, and inflammatory status.

In conclusion, our data together with biochemical analyses^16^ strongly suggest that RANTES filaments can form and be present at physiological conditions *in vivo*. Moreover, that filaments of RANTES can be of functional importance is supported by a study that shows reduced ability of the RANTES tetramer E26A to arrest monocytes on endothelial monolayers^42^. What might be the advantage of presenting RANTES in long filaments on the vascular surface? Clearly, the inherent ability to focus low numbers of molecules for presentation in a patch of concentrated chemokine to rolling leukocytes appears intuitively pleasing. Another, closely related function of RANTES filaments could be to increase the accessibility of the ligand towards the receptor. In fact, Proudfoot *et al*. suggested that oligomerization of chemokines might be important for those whose GAG binding sites overlap with the receptor binding sites, as is the case for RANTES, MCP-1 and MIP-1β so that while some chemokine subunits bind to GAGs others can be exposed to the receptor^13^. The ability to organize into various forms from monomers to long filaments may also generate functionally distinct ligands^7^,^42^,^50^,^51^,^52^. Moreover, it is well documented that chemokines exert effects through dimers or oligomers of G-protein coupled receptors. Although the general view is that the receptor dimerizes shortly after synthesis in the endoplasmic reticulum^53^,^54^, a possible function of the RANTES filaments could be to facilitate ligand binding of two or more receptors. This could increase the number of integrins that are activated and the time for which a leukocyte presents activated integrins, and thereby promote the probability of leukocyte interaction with adhesion molecules and subsequent arrest to the endothelium.

## Methods

### Antibodies and reagents

Fetal bovine serum (FBS), gentamicin, fungizone, L-glutamine, MCDB 131, and Opti-MEM I were purchased from Life Technologies (Paisley, UK), and trypsin-EDTA was from Bio-Whittaker (Walkersville, MD). Recombinant human TNFα and IFNγ, recombinant human epidermal growth factor (EGF), recombinant human basic fibroblast growth factor (bFGF), recombinant human MCP-1, mouse and goat anti-human RANTES antibodies (MAB678, clone 21418, and BAF278, respectively) were purchased from R&D Systems (Abingdon, UK). A second mouse anti-human RANTES antibody was a kind gift from M. Sticherling (Klinikum der Christian-Albrechts-Universität zu Kiel, Germany), and mouse anti-human RANTES clone VL1 was from Biosource (Camarillo, CA). Rabbit anti-human RANTES (500-P36) was from Peprotech (Rocky Hill, NJ), and mouse anti-heparan sulfate, clone 10E4, and hyaluronan binding protein was from Seikagaku Corporation (Tokyo, Japan). The secondary rabbit anti-goat antibody used for electron microscopy was from DAKO (Glostrup, Denmark). The fluorescent (alexa 488 or 594) secondary anti-mouse or anti-rabbit antibodies were from Molecular Probes (PoortGebouw, The Netherlands), fluorescein ulex europaeus agglutinin I, and biotinylated horse anti-mouse IgG from Vector Laboratories (Burlinghame, CA), streptavidin-Cy2 from Amersham Pharmacia Biotech (Piscataway, NJ), and streptavidin-Cy3 from Jackson (West Grove, PA). Protein A coupled to gold particles of different sizes was purchased from George Posthuma (Utrecht, The Netherlands). Heparin was from LeoPharma (Ballerup, Denmark). All other reagents were from Sigma Chemical (St Louis, MO).

### Constructs

RANTES was amplified from cDNA derived from TNFα/IFNγ-activated HUVECs. Primers (forward 5'-CTCTCCCAAGCTTACCATGAAGGTCT-3' and reverse 5'- AGAATCTAGACTAGCTCATCTCCAAAGAGTTGATGTACT-3') were designed to introduce HindIII and XbaI restriction sites (underlined), respectively, that were used to clone the RANTES-encoding DNA fragment into the pcDNA3.1(+) vector (Invitrogen, Carlsbad, CA). Based on this construct, alanine exchange of selected amino acids was performed using the QuikChangeTM Site-Directed Mutagenesis Kit (Stratagene, La Jolla, CA) according to the instructions by the manufacturer. The following primers were used to introduce the mutations, giving the sequence of the sense primers with mutated nucleotides written with small letters: Y3A: 5'-CTGCATCTGCCTCCCCAgcTTCCTCGGACACCACACC-3'; E26A: 5'-CCCGTGCCCACATCAAGGcGTATTTCTACACCAGTGGCAAGTG -3'; 44AANA47: 5'GCAGTCGTCTTTGTCACCgcggcGAACgcCCAAGTGTGTGCCAACC -3'; E66A: 5'- CGGGAGTACATCAACTCTTTGgcGATGAGCGCGGTACCG -3'. The corresponding antisense primers were complement reverse.

### Cells

Umbilical cords were obtained from the Department of Gyneacology and Obstetrics, Oslo University Hospital - Rikshospitalet, with the mothers' written permission, and in accordance with an approved study protocol (Regional Committee for Medical Research Ethics, Health Region South, Norway, Approval S-05152). Human umbilical vein endothelial cells (HUVECs) were isolated as described by Jaffe^55^ and cultured in MCDB 131 containing 7.5% FBS, 10 ng/ml recombinant human EGF, 1 ng/ml recombinant human bFGF, 1 μg/ml hydrocortisone, 50 μg/ml gentamicin, and 250 ng/ml fungizone. The cells were maintained at 37°C in humid 95% air/5% CO_2_ atmosphere and split at ratio 1/3. The cultures were used at passage level one to six.

### Electroporation

HUVECs were trypsinized, washed and resuspended in OptiMEM I containing 2.5% FBS, before transfection by electroporation using 20 μg DNA according to the protocol 0394 from BTX (Holliston, MA). Following electroporation, the cells were cultivated for approximately 24 h before fixation or staining of cell surface associated RANTES.

### Digestion of heparan sulfate

To examine whether RANTES was bound to the surface in a GAG dependent manner, monolayers of HUVECs were stimulated with 10 ng/ml TNFα combined with 1 ng/ml IFNγ for 48 h before incubation with a mixture of heparinase I, II, and III (0.5 U/ml) for 2 h at 30°C and subsequently fixed and labelled for RANTES as described above.

Sterile testicular hyaluronidase (1.0 mg/ml stock in DMEM; type IV; Sigma) or *Streptomyces* hyaluronidase [100 turbidity reducing units (TRU)/ml stock in DMEM; Seikagaku] was added directly to cell cultures to yield a final concentration of 20, 50, or 100 μg/ml and 20 TRU/ml, respectively, and incubation continued for 3 hr at 37°C in a CO_2_-containing atmosphere.

### Adhesion of peripheral blood mononuclear cells

HUVECs were electroporated with wtRANTES and cultivated on gelatine-coated chamberslides (Lab-Tek) for approximately 24 h before addition of 100 ng/ml TNFα (final concentration). After 12 h of stimulation, the cells were cultivated with 200 ng/ml recombinant human MCP-1 for 20 min. The cultures were then washed and peripheral blood mononuclear cells were added to the HUVECs 20 min before fixation. Mononuclear cells were isolated by centrifugation on Lymphoprep according to instructions of the manufacturer (Nycomed, Oslo, Norway).

### Immunostaining protocols and fluorescence microscopy

HUVECs were cultivated on gelatine (1% (w/v)) coated 10 × 10 mm glass coverslips or Lab-Tek chamber slides (Nunc, Roskilde, Denmark). For labelling of permeabilized HUVECs, cells were fixed in 4% paraformaldehyde for 10–15 min before washing in phosphate-buffered saline (PBS). For immunostaining, the fixed monolayers were permeabilized with 0.05% saponin before incubation with the antibodies, alternatively, 0.1% saponin was included in all solutions. In another set of experiments, recombinant RANTES was added to gelatine (1% (w/v)) coated chamber slides in the absence of HUVECs for 35 h. RANTES was incubated in endothelial cell growth medium MCDB 131 without FBS or in conditioned MCDB 131 with FBS. Alternatively, heparin (LeoPharma, Ballerup, Denmark) was added in different concentrations before fixation. After sequentially labelling with primary antibodies and secondary reagents, the slides were mounted in mowiol combined with DABCO or polyvinyl alcohol. Labelling of cell surface-associated RANTES was performed on ice with cold (4 °C) antibody solution. The primary biotinylated antibody was added for 45 min before fixation in paraformaldehyde followed by washing in PBS and sequential labelling with streptavidin-Cy3 or anti-rabbit Cy2. The immunostained cells were examined by an Axioplan 2 imaging Zeiss microscope using Plan-NEOFLUAR 40× and 100× oil-objectives or a confocal laser scanning microscope (Leica TCS, Heidelberg, Germany) with A Plan apochromat 100×/1.4 oil objective equipped with an Ar (488 nm) and a He/Ne (543 and 633 nm) laser. Cells that expressed two or more RANTES filaments, were defined as filament-forming cells in the experiments shown in Fig. 5.

### Cryo-electron microscopy and immunogold labelling

HUVECs were grown in 10 cm diameter culture dishes and cytokine-stimulated for 36 h before fixation in 0.1 M PBS containing 4% paraformaldehyde alone or a combination of 0.1% glutaraldehyde and 4% paraformaldehyde for 3 h at room temperature. After washing in 1 × PBS, cells were scraped-off and spun down. Cell pellets were embedded in 1 × PBS/12% gelatine and after infiltration with 2.3 M sucrose over-night at 4°C, cut into small blocks, mounted on pins and frozen in liquid nitrogen. Ultrathin cryosections of about 60–70 nm thickness were obtained by cutting at −120°C with a Reichert Ultracut S ultracryomicrotome from Leica (Heidelberg, Germany). Cryosections were picked up in a 1:1 mixture of 2% methylcellulose and 2.3 M sucrose. Cryosections were then sequentially incubated with the goat anti-human RANTES antibody, the rabbit anti-goat antibody, and protein A-gold particles diluted in 1 × PBS/1% BSA for 30 min at room temperature with extensive washing between the incubations. (In an alternative immunolabeling protocol, clone ID2/A12 was utilized.) Finally, cryosections were contrasted with a 1:9 mixture of 3% uranyl-acetate and 2% methylcellulose before examination. When quantifying the distribution of RANTES, 30 pictures were utilized and gold particles associated with membrane projections versus the remaining plasma membrane were counted.

### RANTES filaments at flow conditions

Glass coverslips were coated with gelatine (1% (w/v)), HUVECs were added and cultivated at standard conditions for 24 h before stimulation for approximately 40 h with TNFα (10 ng/ml) and IFNγ (1 ng/ml). Next, the cover slips were mounted in a flow chamber which was placed on a microscope stage for live imaging. The stage was enclosed by an incubator with temperature 37 °C and CO_2_ adjusted to 6%. By use of a pump, medium with temperature 37°C was applied to the flow chamber corresponding to shear stress 1 dyne/cm^2^. The flow rate was calculated by use of the formula T = 3μQ/2ba^2^, where T = wall shear stress, μ = coefficient of viscosity (0.7 centipoise), Q = volumetric flow rate (cm^3^/s), a = half channel height (127 × 10^−4^ cm), and b = channel width (0.8 cm)^56^. Confocal images were acquired using an Olympus FluoView1000 inverted microscope with a PlanApo × 60/1.42 oil objective (Olympus, Hamburg, Germany). ImageJ (NIH, Bethesda, MD, USA) and Adobe Photoshop (Adobe systems, San Jose, CA, USA) were used to process and prepare the images.

### Statistical analyses

Statistical analyses were performed by use of GraphPad Prism version 6.04. Responses for each experimental group are presented as means with SEM. Differences between groups were analyzed by using one-way ANOVA with the Šidák method for multiple comparisons. p-values < 0.05 were considered significant.

## Author Contributions

I.Ø., N.B., T.B., A.M.K. performed experiments; I.Ø. and N.B. prepared figures, all authors analyzed data; G.H. initiated the study; I.Ø., N.B., O.B. and G.H. designed the study, I.Ø. and G.H. wrote the manuscript.

## Supplementary Material

Supplementary Information Supplementary figures

## References

[b1] Shamri R. *et al.* Lymphocyte arrest requires instantaneous induction of an extended LFA-1 conformation mediated by endothelium-bound chemokines. Nat Immunol 6, 497–506 (2005). 15834409 10.1038/ni1194

[b2] Bonecchi R. *et al.* Chemokines and chemokine receptors: an overview. Front Biosci (Landmark Ed) 14, 540–51 (2009). 19273084 10.2741/3261

[b3] Anders H. J., Romagnani P. & Mantovani A. Pathomechanisms: homeostatic chemokines in health, tissue regeneration, and progressive diseases. Trends Mol Med 20, 154–65 (2014). 24440002 10.1016/j.molmed.2013.12.002

[b4] Griffith J. W., Sokol C. L. & Luster A. D. Chemokines and chemokine receptors: positioning cells for host defense and immunity. Annu Rev Immunol 32, 659–702 (2014). 24655300 10.1146/annurev-immunol-032713-120145

[b5] Hoogewerf A. J. *et al.* Glycosaminoglycans mediate cell surface oligomerization of chemokines. Biochemistry 36, 13570–8 (1997). 9354625 10.1021/bi971125s

[b6] Kuschert G. S. *et al.* Identification of a glycosaminoglycan binding surface on human interleukin-8. Biochemistry 37, 11193–201 (1998). 9698365 10.1021/bi972867o

[b7] Wang X., Sharp J. S., Handel T. M. & Prestegard J. H. Chemokine oligomerization in cell signaling and migration. Prog Mol Biol Transl Sci 117, 531–78 (2013). 23663982 10.1016/B978-0-12-386931-9.00020-9 PMC3937849

[b8] Middleton J. *et al.* Transcytosis and surface presentation of IL-8 by venular endothelial cells. Cell 91, 385–95 (1997). 9363947 10.1016/s0092-8674(00)80422-5

[b9] Wang L., Fuster M., Sriramarao P. & Esko J. D. Endothelial heparan sulfate deficiency impairs L-selectin- and chemokine-mediated neutrophil trafficking during inflammatory responses. Nat Immunol 6, 902–10 (2005). 16056228 10.1038/ni1233

[b10] Kuschert G. S. *et al.* Glycosaminoglycans interact selectively with chemokines and modulate receptor binding and cellular responses. Biochemistry 38, 12959–68 (1999). 10504268 10.1021/bi990711d

[b11] Proudfoot A. E. *et al.* The BBXB motif of RANTES is the principal site for heparin binding and controls receptor selectivity. J Biol Chem 276, 10620–6 (2001). 11116158 10.1074/jbc.M010867200

[b12] Martin L. *et al.* Structural and functional analysis of the RANTES-glycosaminoglycans interactions. Biochemistry 40, 6303–18 (2001). 11371192 10.1021/bi002670n

[b13] Proudfoot A. E. *et al.* Glycosaminoglycan binding and oligomerization are essential for the in vivo activity of certain chemokines. Proc Natl Acad Sci U S A 100, 1885–90 (2003). 12571364 10.1073/pnas.0334864100 PMC149928

[b14] Johnson Z. *et al.* Interference with heparin binding and oligomerization creates a novel anti-inflammatory strategy targeting the chemokine system. J Immunol 173, 5776–85 (2004). 15494530 10.4049/jimmunol.173.9.5776

[b15] Skelton N. J., Aspiras F., Ogez J. & Schall T. J. Proton NMR assignments and solution conformation of RANTES, a chemokine of the C-C type. Biochemistry 34, 5329–42 (1995). 7537088 10.1021/bi00016a004

[b16] Wang X., Watson C., Sharp J. S., Handel T. M. & Prestegard J. H. Oligomeric structure of the chemokine CCL5/RANTES from NMR, MS, and SAXS data. Structure 19, 1138–48 (2011). 21827949 10.1016/j.str.2011.06.001 PMC3159919

[b17] Blanpain C. *et al.* The core domain of chemokines binds CCR5 extracellular domains while their amino terminus interacts with the transmembrane helix bundle. J Biol Chem 278, 5179–87 (2003). 12466283 10.1074/jbc.M205684200

[b18] Handel T. M., Johnson Z., Crown S. E., Lau E. K. & Proudfoot A. E. Regulation of protein function by glycosaminoglycans--as exemplified by chemokines. Annu Rev Biochem 74, 385–410 (2005). 15952892 10.1146/annurev.biochem.72.121801.161747

[b19] Lau E. K. *et al.* Identification of the glycosaminoglycan binding site of the CC chemokine, MCP-1: implications for structure and function in vivo. J Biol Chem 279, 22294–305 (2004). 15033992 10.1074/jbc.M311224200

[b20] Oynebraten I., Bakke O., Brandtzaeg P., Johansen F. E. & Haraldsen G. Rapid chemokine secretion from endothelial cells originates from 2 distinct compartments. Blood 104, 314–20 (2004). 15044249 10.1182/blood-2003-08-2891

[b21] Oynebraten I. *et al.* Characterization of a novel chemokine-containing storage granule in endothelial cells: evidence for preferential exocytosis mediated by protein kinase A and diacylglycerol. J Immunol 175, 5358–69 (2005). 16210642 10.4049/jimmunol.175.8.5358

[b22] Fujimoto S., Yamamoto K. & Takeshige Y. Electron microscopy of endothelial microvilli of large arteries. Anat Rec 183, 259–65 (1975). 1200402 10.1002/ar.1091830204

[b23] Swensson O., Schubert C., Christophers E. & Schroder J. M. Inflammatory properties of neutrophil-activating protein-1/interleukin 8 (NAP-1/IL-8) in human skin: a light- and electronmicroscopic study. J Invest Dermatol 96, 682–9 (1991). 2022875 10.1111/1523-1747.ep12470606

[b24] Whittall C. *et al.* A chemokine self-presentation mechanism involving formation of endothelial surface microstructures. J Immunol 190, 1725–36 (2013). 23325889 10.4049/jimmunol.1200867 PMC3672850

[b25] Marfaing-Koka A. *et al.* Regulation of the production of the RANTES chemokine by endothelial cells. Synergistic induction by IFN-gamma plus TNF-alpha and inhibition by IL-4 and IL-13. J Immunol 154, 1870–8 (1995). 7530744

[b26] Bao X. *et al.* Endothelial heparan sulfate controls chemokine presentation in recruitment of lymphocytes and dendritic cells to lymph nodes. Immunity 33, 817–29 (2010). 21093315 10.1016/j.immuni.2010.10.018 PMC2996097

[b27] Tanino Y. *et al.* Kinetics of chemokine-glycosaminoglycan interactions control neutrophil migration into the airspaces of the lungs. J Immunol 184, 2677–85 (2010). 20124102 10.4049/jimmunol.0903274 PMC4113427

[b28] Leteux C. *et al.* 10E4 antigen of Scrapie lesions contains an unusual nonsulfated heparan motif. J Biol Chem 276, 12539–45 (2001). 11278655 10.1074/jbc.M010291200

[b29] van den Born J. *et al.* Novel heparan sulfate structures revealed by monoclonal antibodies. J Biol Chem 280, 20516–23 (2005). 15778504 10.1074/jbc.M502065200

[b30] David G., Bai X. M., Van der Schueren B., Cassiman J. J. & Van den Berghe H. Developmental changes in heparan sulfate expression: in situ detection with mAbs. J Cell Biol 119, 961–75 (1992). 1385449 10.1083/jcb.119.4.961 PMC2289686

[b31] Day A. J. & de la Motte C. A. Hyaluronan cross-linking: a protective mechanism in inflammation? Trends Immunol 26, 637–43 (2005). 16214414 10.1016/j.it.2005.09.009

[b32] Appay V., Brown A., Cribbes S., Randle E. & Czaplewski L. G. Aggregation of RANTES is responsible for its inflammatory properties. Characterization of nonaggregating, noninflammatory RANTES mutants. J Biol Chem 274, 27505–12 (1999). 10488085 10.1074/jbc.274.39.27505

[b33] Czaplewski L. G. *et al.* Identification of amino acid residues critical for aggregation of human CC chemokines macrophage inflammatory protein (MIP)-1alpha, MIP-1beta, and RANTES. Characterization of active disaggregated chemokine variants. J Biol Chem 274, 16077–84 (1999). 10347159 10.1074/jbc.274.23.16077

[b34] Shaw J. P. *et al.* The X-ray structure of RANTES: heparin-derived disaccharides allows the rational design of chemokine inhibitors. Structure 12, 2081–93 (2004). 15530372 10.1016/j.str.2004.08.014

[b35] Barreiro O. *et al.* Endothelial adhesion receptors are recruited to adherent leukocytes by inclusion in preformed tetraspanin nanoplatforms. J Cell Biol 183, 527–42 (2008). 18955551 10.1083/jcb.200805076 PMC2575792

[b36] Ley K. & Zhang H. Dances with leukocytes: how tetraspanin-enriched microdomains assemble to form endothelial adhesive platforms. J Cell Biol 183, 375–6 (2008). 18981226 10.1083/jcb.200809173 PMC2575778

[b37] Haraldsen G., Kvale D., Lien B., Farstad I. N. & Brandtzaeg P. Cytokine-regulated expression of E-selectin, intercellular adhesion molecule-1 (ICAM-1), and vascular cell adhesion molecule-1 (VCAM-1) in human microvascular endothelial cells. J Immunol 156, 2558–65 (1996). 8786319

[b38] Carman C. V., Jun C. D., Salas A. & Springer T. A. Endothelial cells proactively form microvilli-like membrane projections upon intercellular adhesion molecule 1 engagement of leukocyte LFA-1. J Immunol 171, 6135–44 (2003). 14634129 10.4049/jimmunol.171.11.6135

[b39] Carman C. V. & Springer T. A. A transmigratory cup in leukocyte diapedesis both through individual vascular endothelial cells and between them. J Cell Biol 167, 377–88 (2004). 15504916 10.1083/jcb.200404129 PMC2172560

[b40] Gerszten R. E. *et al.* Adhesion of monocytes to vascular cell adhesion molecule-1-transduced human endothelial cells: implications for atherogenesis. Circ Res 82, 871–8 (1998). 9580553 10.1161/01.res.82.8.871

[b41] Cinamon G., Shinder V. & Alon R. Shear forces promote lymphocyte migration across vascular endothelium bearing apical chemokines. Nat Immunol 2, 515–22 (2001). 11376338 10.1038/88710

[b42] Baltus T., Weber K. S., Johnson Z., Proudfoot A. E. & Weber C. Oligomerization of RANTES is required for CCR1-mediated arrest but not CCR5-mediated transmigration of leukocytes on inflamed endothelium. Blood 102, 1985–8 (2003). 12763925 10.1182/blood-2003-04-1175

[b43] Braunersreuther V. *et al.* Chemokine CCL5/RANTES inhibition reduces myocardial reperfusion injury in atherosclerotic mice. J Mol Cell Cardiol 48, 789–98 (2010). 19665464 10.1016/j.yjmcc.2009.07.029

[b44] Blanpain C. *et al.* A chimeric MIP-1alpha/RANTES protein demonstrates the use of different regions of the RANTES protein to bind and activate its receptors. J Leukoc Biol 69, 977–85 (2001). 11404385

[b45] Vives R. R., Sadir R., Imberty A., Rencurosi A. & Lortat-Jacob H. A kinetics and modeling study of RANTES(9–68) binding to heparin reveals a mechanism of cooperative oligomerization. Biochemistry 41, 14779–89 (2002). 12475226 10.1021/bi026459i

[b46] Montresor A., Toffali L., Constantin G. & Laudanna C. Chemokines and the signaling modules regulating integrin affinity. Front Immunol 3, 127 (2012). 22654882 10.3389/fimmu.2012.00127 PMC3360201

[b47] Sasaki K., Okouchi Y., Rothkotter H. J. & Pabst R. Ultrastructural localization of the intercellular adhesion molecule (ICAM-1) on the cell surface of high endothelial venules in lymph nodes. Anat Rec 244, 105–11 (1996). 8838428 10.1002/(SICI)1097-0185(199601)244:1<105::AID-AR10>3.0.CO;2-T

[b48] Nakashima Y., Raines E. W., Plump A. S., Breslow J. L. & Ross R. Upregulation of VCAM-1 and ICAM-1 at atherosclerosis-prone sites on the endothelium in the ApoE-deficient mouse. Arterioscler Thromb Vasc Biol 18, 842–51 (1998). 9598845 10.1161/01.atv.18.5.842

[b49] Ren M. *et al.* Polymerization of MIP-1 chemokine (CCL3 and CCL4) and clearance of MIP-1 by insulin-degrading enzyme. EMBO J 29, 3952–66 (2010). 20959807 10.1038/emboj.2010.256 PMC3020635

[b50] Paavola C. D. *et al.* Monomeric monocyte chemoattractant protein-1 (MCP-1) binds and activates the MCP-1 receptor CCR2B. J Biol Chem 273, 33157–65 (1998). 9837883 10.1074/jbc.273.50.33157

[b51] Tan J. H. *et al.* Design and receptor interactions of obligate dimeric mutant of chemokine monocyte chemoattractant protein-1 (MCP-1). J Biol Chem 287, 14692–702 (2012). 22396538 10.1074/jbc.M111.334201 PMC3340267

[b52] Salanga C. L. *et al.* Multiple Glycosaminoglycan-binding Epitopes of Monocyte Chemoattractant Protein-3/CCL7 Enable It to Function as a Non-oligomerizing Chemokine. J Biol Chem 289, 14896–912 (2014). 24727473 10.1074/jbc.M114.547737 PMC4031540

[b53] Issafras H. *et al.* Constitutive agonist-independent CCR5 oligomerization and antibody-mediated clustering occurring at physiological levels of receptors. J Biol Chem 277, 34666–73 (2002). 12089144 10.1074/jbc.M202386200

[b54] Springael J. Y., Urizar E. & Parmentier M. Dimerization of chemokine receptors and its functional consequences. Cytokine Growth Factor Rev 16, 611–23 (2005). 15979374 10.1016/j.cytogfr.2005.05.005

[b55] Jaffe E. A., Nachman R. L., Becker C. G. & Minick C. R. Culture of human endothelial cells derived from umbilical veins. Identification by morphologic and immunologic criteria. J Clin Invest 52, 2745–56 (1973). 4355998 10.1172/JCI107470 PMC302542

[b56] Lawrence M. B., Smith C. W., Eskin S. G. & McIntire L. V. Effect of venous shear stress on CD18-mediated neutrophil adhesion to cultured endothelium. Blood 75, 227–37 (1990). 1967215

