# Supplementary material for: Oligomerized, filamentous surface presentation of RANTES/CCL5 on vascular endothelial cells
Source: Sci Rep. 2015 Mar 20;5:9261. doi: 10.1038/srep09261 (PMC4367157; doi:10.1038/srep09261)
Supplement: Supplementary Information — Supplementary figures [file srep09261-s1.pdf]

SUPPLEMENTARY INFORMATION

Oligomerized, filamentous surface presentation of RANTES/CCL5 on vascular endothelial cells

Inger Øynebråten, Nicolas Barois, Trygve Bergeland, Axel M. Küchler, Oddmund Bakke, and Guttorm Haraldsen

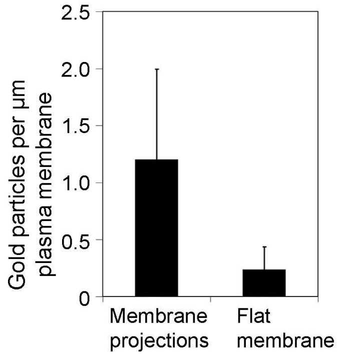

Figure 1S. There was a tendency towards more RANTES labeling on membrane projections compared to the remaining plasma membrane. Cultivated HUVECs were stimulated with 10 ng/ml TNFα + 1 ng/ml IFNγ for 36 h and frozen for cryosectioning before immunogold labeling of RANTES. Quantification of labeled RANTES was based on 30 pictures and by counting gold particles associated with membrane projections and the remaining plasma membrane. The results are expressed as the number of gold particles per µm length of plasma membrane +/-SD. Although there was a tendency of more RANTES on membrane projections than on the remaining plasma membrane, analysis by two-tailed t-test considering $p<0.5$ significant, revealed no significant difference in localization.

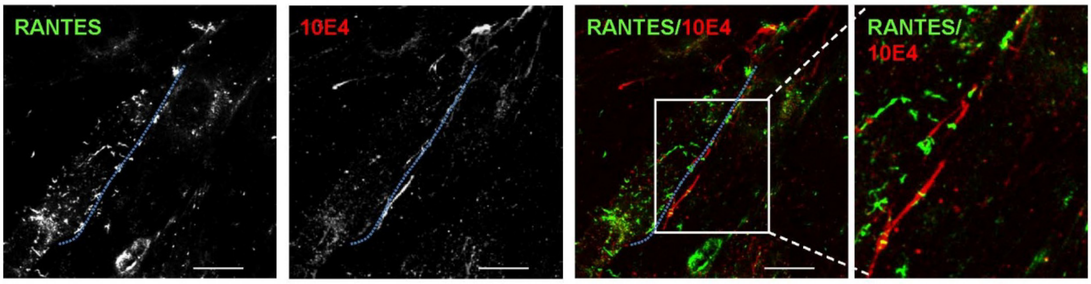

Figure S2. RANTES showed partial co-localization with heparan sulfate organized in thread-like structures. Cultivated HUVECs were stimulated with 10 ng/ml TNFα + 1 ng/ml IFNγ for 35 h, fixed and immunostained with antibodies towards RANTES (rabbit anti-RANTES) and the heparan sulfate epitope 10E4. Scale bar, 10 µm. The blue line indicates the border between adjacent cells. Right image shows high magnification of framed area.
